# The Dangers of Growing Old: Adult Moths Face Higher Predation Pressures than Caterpillars in *Hyles lineata*

**DOI:** 10.3390/insects16040347

**Published:** 2025-03-27

**Authors:** Braulio A. Sanchez, Oceane Da Cunha, Jackson W. Savage, L. Miles Horne, Sol Saenz-Arreola, Kajaya Pollard, Oliver Neria, Spencer Duffendack, Simon Terrazas, Javier M. Diaz, John Deitsch, Brett M. Seymoure

**Affiliations:** 1 Department of Biological Sciences, University of Texas at El Paso, El Paso, TX 79902, USA; basanchez11@utep.edu (B.A.S.);; 2 Chihuahuan Desert Biodiversity REU, Department of Biological Sciences, University of Texas at El Paso, El Paso, TX 79902, USA

**Keywords:** Chihuahuan Desert, Hawk moth, holometabolism, life stage, plasticine models, temporal niche

## Abstract

Holometabolous insects display drastically different morphologies across life stages (i.e., larvae vs. adults). Morphological differences across life stages, such as different sizes and coloration, likely result in differential survival, as predators may find individuals of one life stage more conspicuous and/or more energetically profitable than another. Furthermore, prey conspicuousness may vary temporally because both the sensory environment and predator sensory abilities differ between day and night. Here, we investigated how the interaction between life stage (caterpillar vs. moth) and time of day (day vs. night) influences predation of the white-lined sphinx (Lepidoptera: *Hyles lineata*). We predicted that caterpillars would be less susceptible to predation than adult moths, as adults are larger and have a more conspicuous shape. After quantifying predation for 72 h during dawn and dusk using 199 plasticine replicas each of adults and caterpillars, predation on adult replicas was twice that of predation on caterpillar replicas. Furthermore, replicas were six times more likely to be predated on during the day than during the night. Lastly, attacks were made mainly by birds, which carried out 86% of the attacks on adult models and 85% of those on caterpillar models. These data support the hypothesis that predation rates differ across life stages in holometabolous insects. This research lays a foundation for further investigation into how specific differences in morphology across life stages affect predation and survival in holometabolous insects.

## Simple Summary

Predation on insects can depend upon numerous factors, including morphology, size, age, environment, time, and many others. Most insects undergo complete metamorphosis, in which they begin as eggs, then become larvae, then pupae, and then reproductive adults. Complete metamorphosis has been hypothesized to be advantageous for numerous reasons, including reduced predation between the different stages. Here, we tested the attack rates on a common moth, the white-lined sphinx, in both caterpillar and adult moth stages, using clay replicas. We paired caterpillar and moth replicas in a natural desert environment and monitored attack rates at dawn and dusk for 72 h. By checking attack rates at dawn and dusk, we were able to quantify if the replicas had been attacked during the day or night. We found that most attacks occurred during the day and that the chance of adult moths being attacked was more than twice that of caterpillars. Our study supports the hypothesis that non-adult stages may have reduced predation risk; however, our methodologies are only appropriate for assessing visually guided predators. Thus, future work quantifying predation involving all modalities (e.g., acoustic and chemical) is necessary to fully understand differential predation across life stages in insects.

## 1. Introduction

Of the estimated 1.3 million species of animals, more than 900,000 undergo complete metamorphosis. This speciose group consists of the holometabolous (i.e., endopterygota) insects, such as beetles, moths and butterflies, flies, and wasps. Holometabolism is a form of insect development characterized by having four life stages (egg, larva, pupa, and adult) marked by drastic changes in morphology and lifestyle. These differences between life stages enable larvae and adults to exploit different habitats and specialize in different tasks, such as growth versus reproduction [1,2]. Holometabolism is credited as one of the main life-history traits that has resulted in the immense diversity of insects on the planet [3]. Holometabolism has been suggested to be highly advantageous in part because competition between different developmental stages is reduced [4]. In addition to reduced competition between life stages (i.e., juveniles and adults), holometabolism may also increase survival by reducing predation at important life stages. As only the adult stage can reproduce, the egg, larval instars, and pupa must survive to result in any fitness benefits for the individual. Thus, natural selection may have selected for different adaptations in each life stage, resulting in reduced predation in these non-adult stages. Although differences in morphology are extreme in holometabolous insects, research has mostly focused on only one life stage when investigating predator–prey interactions. Studies have investigated predation pressures on either larval or adult populations [5,6,7]. Predation pressures in *Aedes aegypti* (Culicidae) have resulted in larval stages maturing quicker and reaching larger sizes due to the reduced competition of conspecifics [8]. Furthermore, increased cannibalism has been observed in Ladybird Beetles (Coccinellidae) when predation pressures are absent [9]. Additionally, studies have shown that predators specialize on different life stages of hemimetabolous insects [10]. Unlike holometabolous insects, hemimetabolous insects are characterized by three life stages (egg, nymph, and adult), where the nymphs and adults occupy similar niches and are morphologically similar. Thus, studies showing differential predation on different stages in hemimetabolous insects are mostly showing predation differences due to size. If predators specialize on different life stages in hemimetabolous species, we will suspect even greater predator specialization on holometabolous insects due to the disparate morphologies amongst life stages, such as larval and adult forms.

In holometabolous insects, differences between life stages in morphology, such as coloration and shape, are very drastic. In fact, in most holometabolous species, the juvenile stage and adult stage have minimal resemblance to one another, and unlike hemimetabolous species, the late instars are likely much larger-bodied than the adult. The differences in morphology between life stages in holometabolous insects likely affect predation by visually guided predators such as birds, certain insects, and gleaning bats. In some holometabolous orders, especially Lepidoptera, the most obvious and visually noticeable difference between adults and juveniles is the presence of large conspicuous wings that comprise most of the surface area of the adult. Although many species have evolved very cryptic wings, wings do increase the size of an individual and could affect the search image of a potential predator [11]. Furthermore, wings result in a more obvious body shape when compared to the cylindrical form of many juvenile stages that can more easily be mistaken for non-animate objects, such as twigs or animal feces [12].

As many insects have numerous predators that rely upon different sensory modalities for prey detection, predation risk is likely not consistent across a 24 h period. For many insects, their diurnal predators [13], such as insectivorous birds [14], depend upon visual detection and recognition of their prey, and as light levels begin to decrease, visual predators become less active [15]. Thus, nocturnality can be advantageous for many prey species, as visually guided predators are at a disadvantage [16]. Of course, there are numerous nocturnal predators that do not rely on vision but rely on chemical and acoustic signals [17]. Seifert et al. [6] showed that predation increased during the day and dramatically decreased at night in the Amazonian primary lowland rainforest. Furthermore, predator communities differ between day and night, with the main predators being avian during daylight and arthropods and/or mammals during the night [7,15]. In addition, light conditions affect predator decision making. During low-light conditions, blue tits (*Cyanistes caeruleus*) were shown to increasingly attack the yellow morph of the wood tiger moth (*Arctia plantaginis*)*,* whereas the white morph was attacked more in brighter lighting conditions [18]. Thus, the different life stages of holometabolous insects may have differential predation rates between day and night due to light conditions, the predator community, and predator behavior.

In this study, we investigated the effect of life stage and time of day on attack rates on the holometabolous white-lined sphinx moth (*Hyles lineata*). We predicted that sedentary adult moth replicas (hereafter, “moths”) would be subject to higher predation than sedentary larval replicas (hereafter, “caterpillars”) due to morphological differences, independent of behavioral differences between the two life stages. The adult stage of lepidopterans is likely more conspicuous due to wing shape and size, whereas caterpillars are more likely to masquerade as vegetation (e.g., sticks and leaves). We further hypothesized that the time of day would affect predation, as lighting conditions affect the visual abilities of predators and thus detection and recognition of lepidopteran prey. We predicted that visually guided attacks (i.e., avian attacks) would occur more during the day than at night, whereas non-visually guided predators (i.e., insects) would attack equally between day and night. Lastly, we anticipated finding different predator compositions between the two life stages and times of day. Birds were predicted to be the main predators of both stages during the day, rodent predators to be the main predators at night, and insect predators to be similarly active between both day and night. We tested our predictions by deploying visually realistic replicas of moths and caterpillars during dawn and dusk in the Chihuahuan Desert and then quantifying attacks for 72 h.

## 2. Materials and Methods

### 2.1. Plasticine Replicas

To quantify predation rates between life stages and temporal niches, we constructed plasticine replicas of moths and caterpillars. Plasticine replicas have been used to measure predation on numerous organisms, ranging from reptiles to insects [5,6,19,20,21]. Plasticine replicas enable general identification of the predator: avian predators leave triangular beak marks on the clay, mammalian predators leave teeth impressions, and arthropods leave a variety of marks that range from scratches to single holes (see [15,22] for specifics). Plasticine clay-bodied replicas with paper wings are ideal for studying predation rates on moths, as moths are predominately two-dimensional due to their flat wings. Most moths are palatable and highly profitable prey that spend the majority of each life stage immobile. For example, caterpillars will remain in one location for feeding, whereas moths spend most of their time at rest. Also, plasticine replicas of lepidopteran adults and larvae have been documented to represent accurate predation pressures [22,23]. Lastly, using receptor noise models of predator vision [24], plasticine replicas can match the reflectance properties of wings and bodies, resulting in replicas that are not noticeably different from actual live moths [6,23,25].

#### 2.1.1. Adult Moth Replica Development

**Figure 1:**
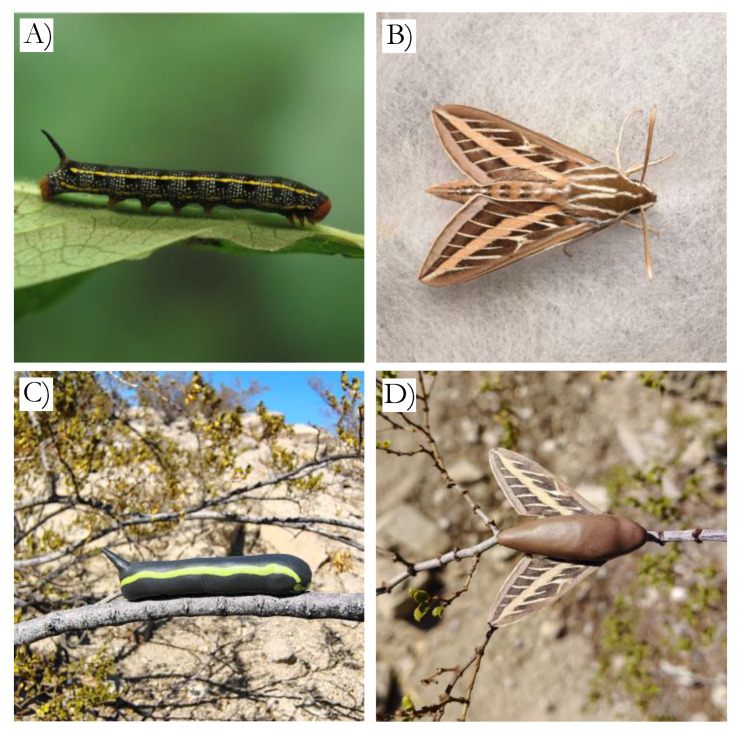
Caterpillar (**A**) and moth (**B**) specimens, along with a caterpillar replica (**C**) and a moth replica (**D**). The replicas in (**C**,**D**) were placed on creosote bushes, as they were in the experiment.

*Hyles lineata*, commonly known as the white-lined sphinx, is the most abundant sphinx moth in North America [26] and is native to the Chihuahuan Desert; see Figure 1. *Hyles lineata* is a large, palatable prey item as both an adult and larva for many insectivorous predators [26]. Moths of *H. lineata* have minimal variation in coloration, with their wing coloration only slightly varying in brightness and hue. For the development of the moth plasticine replicas, eight *H. lineata* moths in pristine condition were randomly selected from the University of Texas at El Paso Biodiversity Collections. To determine the size of the replicas, we calculated the average size by measuring the wing width, wing length, body width, and body length. Then, following [27,28], we quantified wing coloration with a Samsung NX1000 camera (Samsung, Suwon-si, South Korea). As the NX1000 camera is only sensitive to the human-visible wavelengths of light (i.e., 400–700 nm) and as the main predators of moths are passerine birds, which can perceive UV light (i.e., 320–400 nm), we converted the camera from VIS to UV/VIS (i.e., 320–700 nm) by removing the UV/IR filter inside the camera. The camera was then equipped with a Nikon El-Nikkor lens (80 mm; Nikon, Minato City, Tokyo, Japan), a Baader IR/UV cut filter (Baader, Lübeck, Germany), and a Baader Planetarium 1.25” U-Filter to produce visible and UV spectrum pictures (Baader, Lübeck, Germany),. To control light exposure, a UV-suitable gray standard (40% reflectance, Spectralon, Labsphere, North Sutton, NH, USA) was used in every picture.

**Figure 2:**
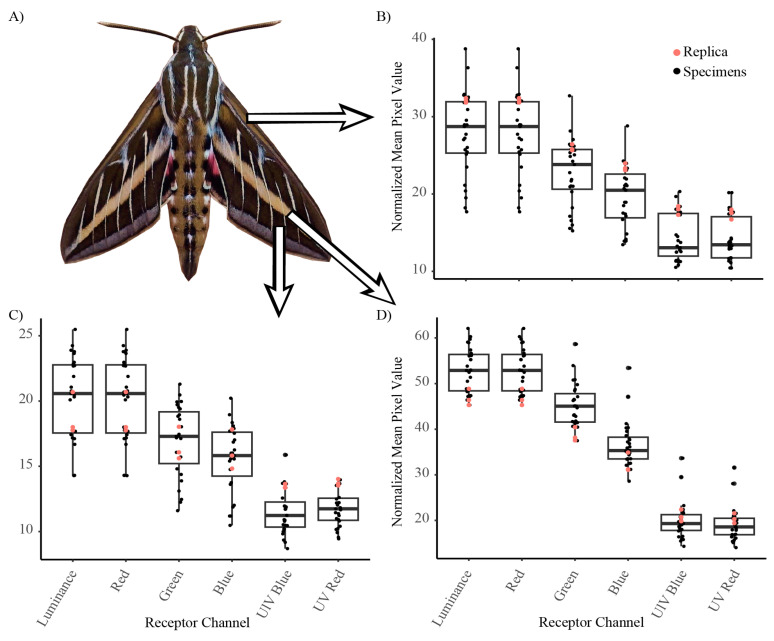
*Hyles lineata*. (**A**) The three locations that were measured for reflectance comparison between the specimens and replicas. The leading edge (**B**), background of wing (**C**), and characteristic white line (**D**) were selected and compared to replica wings. The red dots represent the wings of the replicas, and the black dots represent the specimens. This demonstrated that the replica coloration fell within the natural variability of coloration between individual specimens.

Paper wings that matched the color and pattern of *H. lineata* were printed using a Xerox C310 printer (Xerox, Shinjuku, Japan) and 92 brightness, on white letter Office Depot^®^ multi-use printer paper. We then compared the replicas to moth specimens by photographing both the dorsal and ventral sides of individual moth specimens and paper wings between noon and 1PM under completely clear skies with no obstructions (e.g., trees or buildings). The camera was attached to a tripod and set 56 cm from the lens, ensuring that the camera angle was as perpendicular to the specimen as possible without casting a shadow. Without moving the camera and tripod, we interchanged specimens until all eight individuals and paper wings were photographed. The ISO (800) and aperture were kept constant, but the shutter speed was adjusted between UV (1/4 s) and VIS (1/2000 s) photos, resulting in proper exposures. Then, using the image manipulation program GIMP, we altered the brightness and RGB of three-color patches of the paper wings to better match the spectral reflectance of the moth specimens (Figure 2). We then printed and photographed the revised replica wings repeatedly until the coloration of the wings closely matched the reflectance of the moth specimens (see Figure 1 and Figure 2).

After the color-corrected wings were printed, they were cut, ensuring that only the printed wings were visible and that no trace of the white paper was left. A blank space for the clay body was inserted between the two paper wings (i.e., right and left wings). Four and a half grams of brown Craft Smart oven-baked polymer clay (Craft Smart, Michaels, Irving, Texas, USA) was then molded around the blank space to create the shape of a moth body. However, we first wrapped the white space with plastic wrap to protect the paper wings from plasticizers within the clay, which under high temperatures can be absorbed by the paper and alter the coloration and pattern of the wings (personal observation).

#### 2.1.2. Caterpillar Model Development

Although caterpillars of *H. lineta* have polymorphic coloration that ranges from green to yellow to black [29], we opted to mimic the melanistic morph of *H. lineata*, the body of which is nearly all black with green patterns on the dorsal side, as we have seen this morph at IMRS (personal observation; see Figure 1). The caterpillar body was molded from 4.15 g of black Craft Smart oven-baked polymer clay, and the caterpillar dorsal stripe comprised 0.15 g of green Craft Smart oven-baked polymer clay. The photographic methods described above were not conducted on the caterpillar replicas, as we were constrained by the coloration manufactured by the clay companies. Fortunately, previous studies showed that predators attack even simple clay models [5,30].

### 2.2. Model Deployment and Predation Quantification

During the new moon phase, 26–29 July 2023, we deployed 199 moth models and 199 caterpillar models at the University of Texas at El Paso’s Indio Mountains Research Station (IMRS). IMRS is in southeastern Hudspeth County, roughly 42 km southwest of Van Horn and 5 km north of the Rio Grande, spanning more than 161,000 km^2^. This area of land in the Chihuahuan Desert is mostly undisturbed, with no human settlements within 32 km of its perimeter.

We deployed pairs of the plasticine replicas on 26 July 2023, between 0541 and 0646. One caterpillar replica and one moth replica were placed within two meters of each other on creosote bushes (*Larrea tridentata*) that were one meter in height or taller. Pairs were placed approximately 50 m apart from other replica pairs. Within each pair, the moth and caterpillar models were placed on the top 25% of the same creosote bush on opposite sides, as moths and caterpillars have been seen perching on and eating the upper half of vegetation (personal observation). Both models were placed on branches, tied with Gardener’s Blue Ribbon Sturdy Twists, perpendicular to the ground so that the dorsal side could be seen from above (Figure 1). Replicas were checked every dawn and dusk for attack markings for three days, enabling identification of diurnal or nocturnal attacks. The attacks were categorized as bird, mammal, insect, or unknown based on the indentations left on the clay replicas [30]. Replicas were marked missing when they were not found.

### 2.3. Statistical Analyses

All statistical analyses were run in R version 2023.09.1+494 [31]. Differences in survival probabilities after 72 h between caterpillars and adults were analyzed using Kaplan–Meier survival analysis with the log-rank test ‘survival’ package [32]. Missing models were incorporated into the Kaplan–Meier survival analysis as censored individuals [25,33]. Chi-square analyses were used for testing differences in attack rates between night and day, as well as predator type. As the predation study ran from 26 July through 29 July, when day length was much longer than night length, we corrected for the proportion of daytime and nighttime in calculating the expected numbers of attacks (see [15]). During these 72 h, the average sunrise was at 0616 and average sunset was at 1958, for a total daytime of 13 h and 42 min. However, as we checked the models for attacks during nautical and civil twilight, we equally proportioned twilight between daytime and nighttime by using the average start of civil twilight in the morning (0549) and the average end of civil twilight in the evening (2024). Thus, in our experiment, daytime was on average 14 h and 35 min long and nighttime was 09 h and 20 min long. To adjust for a longer daytime period than nighttime, we multiplied the total number of attacks by the proportion of time that was daytime (0.607) and by the proportion of time that was nighttime (0.393). Lastly, one model was initially thought to have gone missing but was found on the ground with attack marks during a subsequent check, and thus the time of attack was not able to be determined, resulting in 69 models for the temporal analysis. As there were 69 attacks with temporal information, we calculated the number of expected attacks for daytime to be 21 for both the caterpillars and the adults, and we calculated the number of expected attacks for nighttime to be 13.5 for both the caterpillars and the adults.

## 3. Results

**Figure 3:**
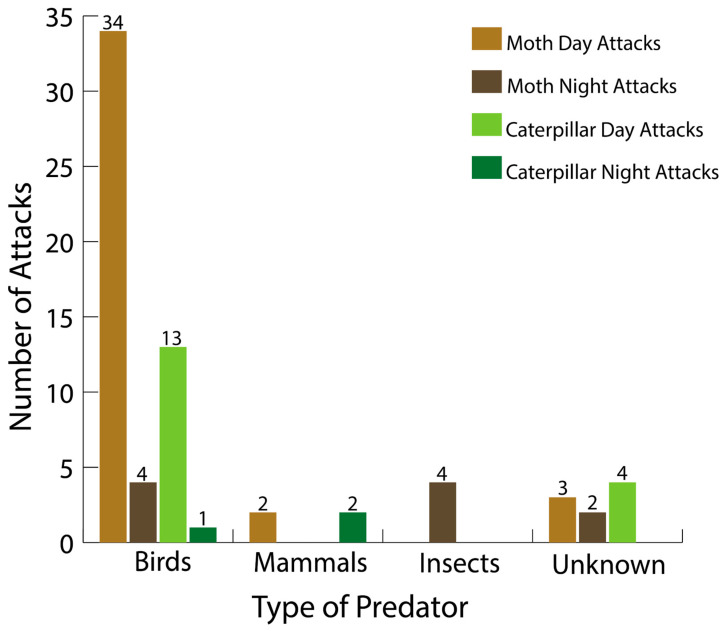
Number of attacks by each predator class during night and day for both caterpillar and adult replicas. The total number of attacks depicted is 69, as 1 unknown attack could not be categorized as occurring during the day or night; see text.

Of the 398 models deployed in the field, 70 (17.5%) were attacked and 30 (7.8%) went missing (Table 1 and Table 2). Of the 70 attacked models, 50 were adults and 20 were caterpillars (Table 1). Of the 30 missing models, 13 were adults and 17 were caterpillars (Table 2). Birds were the primary predators (74% of attacks), and mammals and insects were each subject to 5.7% of attacks (Table 1 and Figure 3). Fourteen percent of the attacks were too ambiguous for predator identification and were classified as unknown. Birds were the primary predators of both the moth and caterpillar replicas, 76% and 70%, respectively. Of the 69 attacked models with temporal information, 60 were attacked during the day and 9 were attacked at night (Table 1). Of the 30 missing models, 20 were lost during the day and 10 at night (Table 2). Of the 20 attacked caterpillars, 17 were attacked during the day. Of the 49 attacked moths, 43 were attacked during the day.

**Figure 4:**
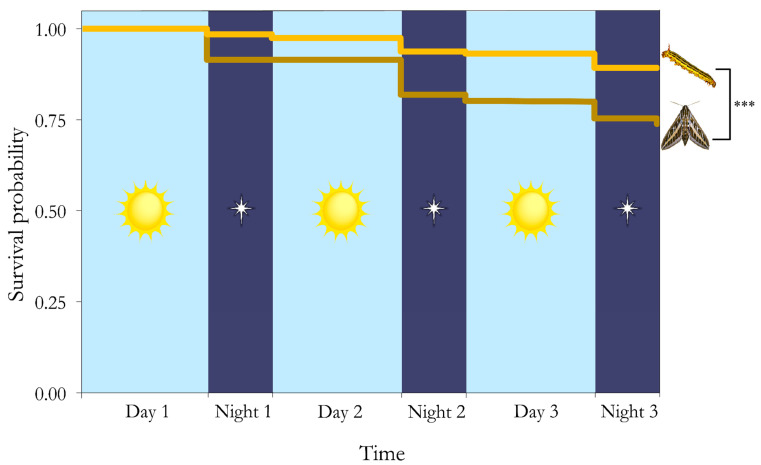
The survival probabilities of clay replicas through time divided into intervals of day and night. Caterpillars had much higher survival compared to moths, and both life stages had higher predation during the daytime than at night. As day length was 14 h and 35 min and night length was 9 h and 25 min, the figure shows shorter durations of nighttime than daytime. *** represents significance of log-rank test, *p* < 0.001.

Survival analysis revealed that caterpillars had higher survival than moths (log-rank, Χ^2^ = 16.1, df = 1, *p* < 0.001; Figure 4). The attacks significantly differed between life stage and time of day (Χ^2^ = 36.14, df = 3, *p* < 0.001; Figure 3 and Figure 4). There were significantly more attacks on adults during the day than at night (Χ^2^ = 14.53, df = 1, *p* < 0.001; Figure 3 and Figure 4). And there were significantly more attacks on caterpillars during the day than at night (Χ^2^ = 5.21, df = 1, *p* = 0.022; Figure 3 and Figure 4). Predator composition also varied significantly, with most attacks being made by birds (Χ^2^ = 76.8, df = 2, *p* < 0.001; Figure 3).

## 4. Discussion

We tested for differential predation between life stages in a palatable moth species and found support for the hypothesis that predation on holometabolous insects varies depending upon life stage, time of day, and predator composition. Adults were subject to much higher predation than caterpillars during the day, whereas there was no difference in predation between life stages at night. Also, birds were overwhelmingly the main predators of both adults and caterpillars, but insect predators appeared to only attack moths and not caterpillars. However, as these findings were obtained with sedentary plasticine models, we can only interpret these findings for visually guided predators and sedentary insect prey.

Differences in predation between life stages in holometabolous insects has been an understudied area of research. Previous research on predation rates in holometabolous insects has focused on one life stage and not both. Comparing attack rates between adults and juveniles from different studies is not prudent, as these studies may have involved differing questions, species, and environmental conditions. Many predation studies on adult holometabolous insects focus on aposematic species or polymorphisms [34,35]. In previous studies, predation rates on adult lepidopterans ranged from around 3% in cryptic tropical butterflies [34] to more than 30% in palatable moths in the desert [15]. Previous studies on juvenile holometabolous insects have mostly focused on clay replicas of caterpillars and found similar variation in attack rates, ranging from around 1% up to 26% [7,36,37,38]. However, as all these studies investigated questions pertaining to aposematism, coloration, and overall trophic ecology, these attack rates are not reliable for comparing between life stages. Here, we found an attack rate of 27% for moths and 11% for caterpillars when directly comparing the two life stages. These rates are within the range of the previously reported attack rates, and thus these attack rates are likely accurate estimations of attack rates on moths and caterpillars in the field. From this study, we conclude that adults are at higher risk of predation than juveniles.

We also showed that both life stages are more susceptible to predation during the day than at night, supporting the hypothesis that nocturnality is advantageous for species to avoid diurnal predators [16]. Our result showing that 85% of attack rates occurred during the day supports the hypothesis that visually guided predators are more active during the day and/or have enhanced vision for detecting and recognizing prey. These results are in line with previous work on temporal niches and predation. Seifert et al. [39] showed decreased predation at night on caterpillar replicas in both closed-canopy and tree-fall gaps, with over 12% more attacks happening during the day when compared to the night. Additionally, previous work on the adult stage of *H. lineata* showed that survival was almost seven times higher at night regardless of habitat type (i.e., urban or rural) [15].

Although mammals and insects attacked the replicas, the main predators in our experiment were birds. The mammals that attacked both the moths and the caterpillars were likely rodents, whereas identifying the insects that attacked the moth replicas is more challenging. It is likely that the moths were attacked by hemipterans, coleopterans, and hymenopterans. Although interesting to note, the insect attack rates on only moth replicas may not be biologically meaningful, as adult moths are likely to fly away if they begin to be attacked by ants or other predaceous insects. As birds were the main predators, the hypothesis that visually guided predators are the main predators of sedentary prey is supported. Bird species are tetrachromatic, enabling high color-rendering abilities under bright conditions [24,40,41]. Not surprisingly, most of the attacks were made during the day, when insectivorous birds’ color vision is most reliable [14]. Due to our experimental setup, chemically and auditorily guided predators were not represented in our experimental design, as our models were sedentary and did not emit sounds or chemical cues that are specific to *H. lineata* [42,43,44]. For example, aerial hawking bats are well-known predators of moths and rely on echolocation to be able to detect their prey when they are in flight [44]. Furthermore, substrate-gleaning bats, which have better vision, still rely on auditory cues to be able to spot prey perched on vegetation, such as caterpillars [45]. Parasitoid wasps, another predator of caterpillars, are able to cue into chemical signals emitted by plants when being eaten by insects [46,47,48]. Furthermore, parasitoid wasps have also been shown to be able to find their prey when exposed to the feces of caterpillars by associating and recognizing the chemicals contained in the caterpillars’ feces [46]. Lastly, there were visual cues that predators would not have been able to observe given our experimental design, such as leaf damage on vegetation resulting from herbivorous caterpillars [49]. Thus, we cannot be certain that all predators of *H. lineata* were represented, suggesting that predation rates may have been underestimated.

Visual predators also rely on search images to detect prey. For example, birds discriminate and look for familiar rather than novel prey [50]. Our study suggests that visual predators possibly have a stronger search image for moths than they do for caterpillars of *Hyles lineata.* The moths of *Hyles lineata* are present from spring to fall and show little variation in coloration and pattern. Unlike moths, their caterpillar stage shows immense variation in color, ranging from green and yellow to black [26,29]. This could explain the higher predation rates on moths compared to caterpillars. In addition, black morphs have been linked to shorter photoperiods [29], and our experiment was conducted during the summer, which has the longest photoperiods of the year. Predators may have a seasonal search image, such that they will look for melanistic *H. lineata* caterpillars during the fall and winter months but not during the summer months, as predators have been shown to discriminate between morphs [51,52]. Consequently, the morph chosen could explain the lower attack rates on the caterpillar replicas, our replicas being modeled after the melanistic form of *H. lineata*. Again, we chose the melanistic form as we have seen this morph at the study site, whereas other morphs have not been seen. However, no assessment of the relative abundance of different morphs across seasons exists, and future research is necessary to better understand the effects of different morphs on predation rates. Additionally, our replicas were a spectral match to the moth specimens, but our caterpillar replicas were not specifically matched to the reflectance properties of caterpillar specimens. Matching clay spectral reflectance was not possible due to the very limited options of clay colors commercially available. This could explain the higher predation rates of the moth replicas compared to the caterpillar replicas, although various similar studies have used simple clay caterpillars where spectral matching was not performed and high attack rates were observed [5,30,36]. Furthermore, the black color morph of the caterpillar replicas may have been the reason for lower predation, as clay color has been shown to affect predation rates, with some clay colors like green and terracotta resulting in higher predation than gray and brown clays [37].

This study was conducted during three days during the monsoon season in west Texas. Thus, this study offers a brief snap-shot of predation pressures on Hyles lineata during a time when both caterpillars and adults are active [53]. Hyles lineata shows a bimodal abundance curve for both caterpillars and adults, one peak in spring and another in late summer [53]. Thus, we anticipated that predation pressures would be higher during the testing period. The monsoon season also coincides with an increase in vegetation in the Chihuahuan Desert, which also results in greater animal activity. However, the findings here are only representative of this wet season in the Chihuahuan Desert, and the predation pressures between larvae and adults may be drastically different during the early spring, when caterpillars are most abundant, or during late fall, when moths are most abundant. Future work investigating the effects of seasonality on differential predation of holometabolous insects is needed.

Predator–prey interactions in holometabolous insects have been underrepresented in the literature, as the larval and adult stages are often treated as entities separate from each other. Using Hyles lineata, an abundant holometabolous insect in the United States, we aimed to find support for the idea that there are differences in predation depending on developmental stage in holometabolous insects. The results showed greater predation on the moth stage compared to the caterpillar stage, demonstrating that studies testing predator–prey interactions in holometabolous insects should not dismiss the developmental stage. Although clay-replica predation experiments have mainly focused on aposematic adults, clay replicas are also appropriate for palatable species across life stages. Future work should expand on differential predation across life stages in Endopterygota, as the great majority of biodiversity comprises holometabolous insects.

## Figures and Tables

**Table 1:** Number of attacks recorded by type of predator. Categories include birds, mammals, insects, and unknown. The type of predator was determined by the type of indentation left on the plasticine models. Unknowns refer to replicas that had indentations that could not be categorized as bird, mammal, or insect. Missing replicas were not found in their locations but could have been attacked and removed by a predator or removed by non-relevant forces such as wind or pedestrians.

	Caterpillars Attacked	Moths Attacked	Total Attacks
Night	3	6	9
Day	17	43	60
Bird	14	38	52
Mammal	2	2	4
Insect	0	4	4
Unknown	4	6	10
Total	20	50	70

**Table 2:** Number of missing replicas categorized by life stage and time.

Missing	Caterpillars Missing	Moths Missing	Total Missing
Night	4	6	10
Day	13	7	20
Total	17	13	30

## Data Availability

The predation data are freely available at Brett Seymoure’s Mendeley Data Repository.
